# Development and Implementation of a Total Worker Health^®^ Mentoring Program in a Correctional Workforce

**DOI:** 10.3390/ijerph18168712

**Published:** 2021-08-18

**Authors:** Sara Namazi, Rajashree Kotejoshyer, Dana Farr, Robert A. Henning, Diana C. Tubbs, Alicia G. Dugan, Mazen El Ghaziri, Martin Cherniack

**Affiliations:** 1 Department of Health Sciences, Springfield College, Springfield, MA 01109, USA; 2 Department of Occupational and Environmental Medicine, University of Connecticut Health Center, Farmington, CT 06030, USA; rkotejoshyer@uchc.edu (R.K.); dana@danafarrphd.com (D.F.); adugan@uchc.edu (A.G.D.); cherniack@uchc.edu (M.C.); 3 Department of Psychological Sciences, University of Connecticut, Storrs, CT 06269, USA; robert.henning@uconn.edu (R.A.H.); dianatubbs@gmail.com (D.C.T.); 4 Susan and Alan Solomont School of Nursing, University of Massachusetts Lowell, Lowell, MA 01854, USA; Mazen_ElGhaziri@uml.edu

**Keywords:** total worker health, correctional workforce, health mentoring, workplace wellness, occupational safety

## Abstract

Correctional officers (COs) are exposed to a number of occupational stressors, and their health declines early in their job tenure. Interventions designed to prevent early decline in CO health are limited. This article describes the development, implementation, and evaluation of a one-year peer health mentoring program (HMP) guided by Total Worker Health^®^ principles and using a participatory action research to collectively address worker safety, health, and well-being of newly hired COs. The HMP aimed to provide new COs with emotional and tangible forms of support during their first year of employment, including peer coaching to prevent early decline in physical fitness and health. The development and implementation of the HMP occurred across five main steps: (1) participatory design focus groups with key stakeholders; (2) adaptation of an existing mentoring handbook and training methods; (3) development of mentor–mentee recruitment criteria and assignment; (4) designing assessment tools; and (5) the initiation of a mentor oversight committee consisting of union leadership, corrections management, and research staff. Correctional employee engagement in the design and implementation process proved to be efficacious in the implementation and adaptation of the program by staff. Support for the HMP remained high as program evaluation efforts continued.

## 1. Introduction

Correctional officers (COs) are front-line employees who work in prisons and jails and are exposed to many unique risks that can jeopardize their safety and well-being [1,2,3]. COs have one of the poorest health profiles of any public safety occupation, and their health declines early in their job tenure [1,4]. CO life expectancy is also affected by job-related stressors and is below the national average (59 versus 75, respectively) [5]. Working as a CO involves extended and irregular shift work [6,7], demanding interpersonal interactions, and repeated exposure to verbal and physical trauma [8,9]. Responses to stressors experienced by COs on the job can spill over to their lives outside of work and can be a source of work–family conflict [10,11]. If COs do not develop healthy coping behaviors early in their careers, the demands of their work can have long-term adverse effects on their health and overall well-being [12]. However, interventions designed to help protect and promote CO health and well-being in the early stages of COs’ careers are scarce [1,13,14]. Furthermore, because the risks to COs are widespread and, therefore, systemic, an individualized psychological approach is likely to be incomplete in addressing the root causes of poor health [10].

Health Improvement Through Employee Control (HITEC) II, currently in its third phase, was a partnership with the Department of Correction (DOC) in a Northeastern state that was formed to develop, implement, and evaluate interventions intended to improve the health, safety, and well-being of correctional employees [1,4]. The Total Worker Health^®^ initiative of the National Institute for Occupational Safety and Health (NIOSH) recognizes work as a social determinant of health and aims to protect the safety and health of workers, and advance their well-being by creating safer and healthier work [15]. HITEC II used a participatory action research (PAR) approach that engaged front-line workers in identifying and addressing health issues through their direct involvement in the design, implementation, and evaluation of interventions specific to their health and safety needs [13]. Further, the HITEC II approach is consistent with Total Worker Health principles and practices because interventions are designed to address risks arising from both the physical and psychosocial work environment, and risks from outside of the workplace [1,2].

In a preliminary study of 326 COs conducted in 2008, HITEC research staff found that CO health declines rapidly early in work tenure [1]. Despite being relatively young with a mean age of 41 (SD = 7.2) years, the population sampled was in relatively poor health. Eighty-five percent (85%) were overweight or obese (BMI of >30); 35% were pre-hypertensive and 56% were hypertensive, and 31% reported symptoms of clinical depression [1,2]. These observations emphasize the need for preventive workplace health practices to improve physical and psychosocial health of COs early in their career.

In the past decade, the public safety sector has applied and recommended peer mentoring to facilitate an easier transition for new recruits on the job [16,17]. Through peer mentoring, new COs can learn to anticipate, identify, and manage the health risks related to their jobs from experienced co-workers. Peer mentoring can also help these new recruits handle risks that arise from the physically dangerous aspects of corrections work, which can have long-term health consequences for employees [18]. Additionally, mentees with high-quality traditional mentoring relationships show a more favorable level of organizational commitment than mentees with poor or fair quality relationships [19,20,21]. However, there is a lack of evidence on health peer mentoring programs in general, and specifically within corrections.

Recognizing the potential decline of CO health during the first years of employment, HITEC II research staff partnered with union leadership, line-level staff, and managers in the DOC in the development, implementation, and initial assessment of a one-year peer-to-peer health mentoring program (HMP) for newly hired COs (newly hired COs who are receiving training are also known as cadets) using a Total Worker Health participatory approach. The idea of developing a HMP came directly from the three regional union leaders representing line-level staff. Union leaders were concerned about the health of their members and recognized that newly hired COs may be less resistant to on-the-job training and preventative health programs, compared to more tenured staff.

The HMP concept can be contrasted with traditional workplace mentoring programs that largely focus on supervisors guiding or enhancing junior employees’ (mentees) personal and career development, where there is a large gap between short-term on-the-job mentoring and later selection for eventual promotion [22]. Despite these programmatic differences, there is ample evidence that employees who receive traditional supervisor or senior employee-centered workplace mentoring benefit in terms of confidence on the job, satisfaction with their jobs, higher incomes, and better job retention [23,24,25].

The main goals of the HMP were to help newly hired COs develop positive health behaviors, and to provide them with peer coaching on health topics to prevent early decline in physical fitness and promote healthy eating, stress management, and work–family balance. In this article, we describe the five steps that were used to design and implement the HMP.

## 2. Methods

**Figure 1:**
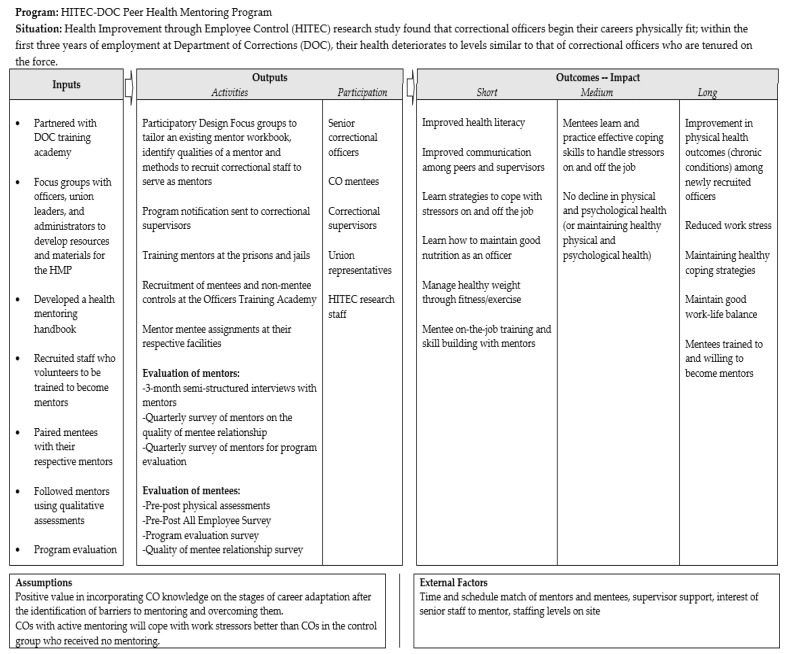
Logic model depicting the process of the Health Mentoring Program (HMP) for newly hired correctional officers (COs).

The HMP was developed in five major steps (design, adaptation, recruitment, assessment, oversight), as detailed below. The overall process and design of the HMP is depicted in a logic model (Figure 1). The program was approved by the university institutional review board.

A core planning group met regularly during the development of the HMP (approximately a total of six meetings per month) and consisted of research staff and union presidents for line-level correctional staff and some of their designees. The meetings focused on the development of the mentoring program and review of draft materials. Additionally, a series of participatory design focus groups were organized with correctional officers who potentially could become mentors. The participatory design focus groups were conducted differently than conventional focus groups and were used to inform ongoing design and development of the HMP and associated materials. Summary findings of suggestions from these participatory focus groups were immediately reported back to the core planning group by research staff in order to inform ongoing program design efforts.

## 3. Results

The following results highlight the process of development and implementation of the HMP, which occurred across the following five main steps:

Step 1. Designing the HMP through Participatory Design Focus Groups.

In line with PAR techniques, the participatory design focus groups provided the means for engaging DOC staff as equal partners in all aspects of program design and implementation [13,26,27]. A guide to the participatory design focus groups was developed based on findings from our previous study of health issues and concerns among corrections workers at two prisons in the Northeast [2]. This guide was used as a reference point in the development of the HMP. More specifically, focus group participants were asked to provide input regarding the following aspects of HMP design: setting program goals; qualities of an effective mentor; salutogenic activities that would strengthen mentor participation [28]; adaptation of an existing mentor workbook and training program; creation of mentor recruitment criteria and a recruitment protocol; formulation of a data collection plan on mentoring activities and on mentee outcomes; and assignment of a mentor oversight committee.

Focus group participants were chosen based on their tenure and rank at DOC. Specifically, participants had to have at least 5 years on the force and prior experience in training newly hired officers on the job. One participatory design focus group consisted of members of the supervisory staff (captains and lieutenants) and two participatory design focus groups consisted of COs, representing two of the three union locals in the state. Participatory design focus groups met at local union offices; 23 officers and five captains and lieutenants participated.

Participatory Design Focus Group Findings: There were differences between the groups in their recommended weighting of experience and personal qualities of prospective mentors, but there were also noted differences of this weighting among members of each participatory design focus group. For example, some considered interpersonal qualities to be more important than technical skills in prospective mentors. The interpersonal skills identified as significant were as follows: *someone to whom one can speak freely, someone who sets a good example, and someone who demonstrates good work quality and good communication skills*. Confidence was expressed among all participatory design focus groups that a well-conceived mentor training program could be developed and sustained. They also felt that the training should be jointly designed with the research team, COs, and Training Academy personnel (the Training Academy has a dedicated site for training newly hired COs and also for providing ongoing training within the DOC).

Although all such ideas gathered in the participatory design focus groups were carefully considered, some of the ideas, although desirable, would have faced structural obstacles if implemented. For example, there was a strong desire for all mentor training to occur at the Training Academy, and for mentors to meet each other monthly in order to share the knowledge gained. However, these ideas proved infeasible due to the geographic dispersion of the correctional facilities and staffing limitations for coverage of those who would be meeting. The participatory design focus group members also recommended matching the gender of mentor and mentee because of the specific challenges to women in the male-dominated workforce but this was not always possible. One unforeseen barrier identified by the participatory design focus groups involved the tenure requirement for mentors and how this would affect shift alignment. In the participatory design focus groups, it was contended that to be a mentor, a CO needed at least 7 years of tenure and a disciplinary record that was currently clear. It was argued that mentor/mentee pairs would not need to be limited to working the same hours, and that they could instead work on adjacent shifts and meet at shift change. However, the arrival of the first mentees revealed this was an unworkable plan when it became apparent that most new officers worked the third shift whereas officers with 7 years of tenure worked the first shift. The SWSC offered a solution to this problem, which was to reduce the tenure requirement to 3 years, allowing for more within-shift mentoring to occur. One principle that could not be similarly compromised by the SWSC was the provision that mentor supervision would be handled by line-level COs, rather than supervisory staff. An important participatory design focus group recommendation was that, although supervisory staff could participate in establishing formal mentor selection criteria, the actual selection of mentors needed to fall under the authority of the COs. One remaining concern that needed to be addressed was the possibility that a mentor might be contested by management because of an undisclosed disciplinary action that was pending, or investigation of potential criminal activity that was ongoing. As the program was further developed through participatory design over time, resolutions on such process issues were worked out within labor leadership meetings. A management oversight committee structure that was proposed and eventually adopted was found to be effectively preventive; it had yet to be convened to resolve a labor-management conflict at the time of this writing.

Step 2. Adaptation of an Existing Mentoring Handbook and Training.

Building on the information generated from the participatory design focus groups, the research team and frontline COs modified an existing career mentoring workbook from the Southern Region of the National Institute of Corrections Academy Division’s Regionalization Project of 2002 [29]. The resulting new handbook, “Health Mentoring in the Correctional Workplace”, consisted of eight sections, entitled: (1) What is Mentoring? (i.e., performance objectives, mentors vs. supervisors, resources, rules of mentoring); (2) Listening skills; (3) Basics of Mentoring (i.e., setting expectations, checking progress); (4) Giving Feedback (advice on ways to engage in regular exercise despite a challenging shift schedule, making healthy food choices at work and home, stress management, and addressing work-family issues); (5) Avoiding Pitfalls, such as conflict between individual health peer consultations and upper level administrative prerogatives; (6) Transitioning and Special Topics (i.e., mentoring and the generation gap; what mentors can do to prevent suicide; incivility); (7) Guidelines for Sessions; and (8) Forms.

The subsequent training of mentors by HITEC II research staff relied on several approaches tailored to adult learners. A presentation using Microsoft PowerPoint was developed that included both lessons from the Handbook and interactive scenarios and scripts. To accommodate agency stipulations on educational travel and release time, the training session was developed to last 1 to 1.5 h with provision for later in-person follow-up. Training delivery at a centralized facility was not feasible due to the geographic distribution of mentors across 18 facilities. Consequently, training sessions were scheduled at each correctional facility during normally assigned shifts. In addition to the Handbook, mentors received a binder that included the initial goal-setting contract, record keeping forms, and materials for quarterly evaluation of their mentee’s personal development and career goals.

Step 3. Mentor–Mentee Recruitment Criteria and Assignment.

An important consensus recommendation from earlier participatory design focus groups was that supervisory staff could participate in establishing formal mentor selection criteria and in making mentor recommendations, but that the actual selection of mentors should fall under the authority of the COs. Focus group participants had also recommended that mentor supervision should not be handled by supervisory staff.

COs working in the cadets’ destination facilities were first introduced to the HMP and the role of mentors at roll call, a regular event that is held at the start of all shifts. Research staff delivered a short presentation on the purpose of the HMP program, on the desired qualifications for mentorship, and on mentors’ obligations and their expected time commitment. Interested prospective mentors signed up either with research staff, through a confidential channel at onsite locations, or via e-mail. Requirements to become a mentor included having worked as a CO for at least 5 years (later reduced to 3) and having remained in good disciplinary standing with the DOC. Because of the unexpectedly high level of interest on the part of mentor candidates, union leadership regularly reviewed individual qualifications, although rejection was uncommon.

To help avoid treatment bias and minimize the effects of a wide range of confounding variables, successive cadet classes were recruited into either the Personalized Follow-up Program (PFP) as the mentored group, or into the Standard Follow-up Program (SFP) as the non-mentored control group. Those in the SFP received conventional on-the-job training (OJT) that all new cadets receive upon transfer to a facility. In the project years 2013–2014, there were five classes matriculating at the Officers’ Training Academy, the inception point for new officers. Three of these classes were assigned to the PFP condition, and two were assigned to the SFP condition. All cadets in each class were invited to participate in the study, as either mentees or controls depending on the inception class, or to decline the invitation without consequence. Those choosing to participate were consented into the study.

Among 406 eligible cadets in the PFP classes, 183 (45.1%) elected to participate in the study. Thirteen corrections facilities within the state had mentors and mentees in the HMP (Table 1). Four facilities receiving the fewest number of mentees are combined in Table 1 because there were few mentor–mentee pairs (*n* = 11).

Participants completed the baseline survey and physical assessment. The ratio of mentors to mentees ranged between 1:1 and 1:3. The majority of mentees were male (76.4%), which was representative in this working population of officers. The results from the surveys and physical assessments at baseline and follow-ups are presented in a separate article reporting on the efficacy of the program [30].

Mentees were assigned to facilities after their academy-based orientation and training was completed. Their orientation and training included a brief internship placement at a facility that may have been different from their final (destination) facility. Research staff assigned mentors to each mentee following their graduation from the training academy and being posted at their destination facility. Mentors and mentees working the same shifts were paired to allow and optimize scheduled meetings. Supervisors at the designated facilities provided relief time for these on-site mentor–mentee meetings. Following mentor–mentee assignments, study staff conducted follow-ups, usually one week later, to ensure that initial contact between mentors and mentees had occurred successfully. These and subsequent follow-ups were initiated through reminder e-mails and phone calls with the mentors. Study staff also sent mentors a reminder e-mail that reiterated the importance of the record-keeping process for study integrity. Additionally, study staff visited each facility every three months to conduct a scheduled follow-up with the mentors that included refresher training with review of the existing materials from the initial training. To support further communications and provide technical assistance, there was also ongoing phone and internet access to the study team that was made available throughout this period.

Step 4. Health Mentor Program Assessment.

At intake and at one-year post-assignment, all participants in both the PFP arm (mentored group, *n* = 183) and in the conventional SFP arm (non-mentored group, *n* = 86) completed a survey and were offered a brief physical exam on a voluntary basis. Based on the initial follow-up results, and previously existing studies on declining health of COs, it was evident and necessary to evaluate the mentee outcomes after around 3–5 years of employment [1,3]. Accordingly, a long-term evaluation of mentee health outcomes was planned at the end of 5 years of their employment by repeating the physical assessment and survey measures among participants. The physical assessment consisted of height, weight, blood pressure, and body fat percentage using the bioelectrical impedance method [11,31]. Participants completed HITEC’s All-Employee Survey (AES) at baseline and after at least one year of receiving health mentoring. This comprehensive survey is a generic CPH-NEW instrument that can be modified to particular conditions of employment [31]. It assesses safety, physical and mental health, and well-being, in addition to several job design and workplace factors [2,11]. The AES had already been adapted in 2008 for the corrections workforce and its results, as noted earlier, were influential to the mentoring project [1]. Participants completed a modified and shortened version of the AES at baseline because many of the job-specific domains in the AES would have been muted by the absence of prior employment at the DOC. Baseline surveys and examinations were completed at a single site, the Officers Training Academy. The follow-up surveys and physical examinations were conducted a year later at the participants’ assigned facilities. The follow-up survey differed from its baseline predecessor because it now included employment-related questions modified for corrections, with some specific additions. Both groups also had access to newsletters and a website with health information tailored to corrections work.

The research staff held onsite quarterly meetings with mentors which lasted about 20–30 min. Semi-structured interview guides created by HITEC II research staff and vetted by the participatory design focus group participants were used to assess the quality of the mentor–mentee relationship. These semi-structured interviews with mentors were designed to collect information on mentoring progress, frequency, type of meetings (formal or informal), topics discussed, health goals, perceptions of mentoring, and barriers and facilitators of peer health mentoring. The quality of the mentor–mentee relationship, and the frequency and quality of mentoring, was also assessed with a survey with mentees during their evaluation at the end of the one-year HMP. The aim of the process was to evaluate the quality of relationship overlaps and discrepancies with dual source data.

Step 5. Mentoring Oversight Committee.

Two different groups, separated by chronology, were charged with oversight of the HMP. In the first 2 years of the program, the major impetus came from regular meetings with the bargaining unit presidents and their representatives. The HMP was one of two major projects generated by HITEC II, and a Study Wide Steering Committee (SWSC) which was in place from 2007, beginning with HITEC I [1]. The SWSC consisted of key stakeholders, such as the HITEC II research team, union leaders, and DOC administration (Director of Human Resources, Deputy Commissioner, and Commissioner). Because the SWSC was developed as an oversight platform for all HITEC-related work, and given its labor and management representation, it assumed oversite of the HMP once the program was established. The SWSC maintained final authority over HMP practices, assisting in implementation by scheduling mentoring time into the workday through coordinating with facility supervisors, and by scheduling times for focus groups, evaluation, surveys, and testing. Consistent with PAR principles, research activities and program activities were discussed, approved, and reviewed at SWSC meetings, which have occurred every 2–3 months over the past 10 years. By agreement, all evaluation results, once stripped of potential personal identifiers, were made available to the SWSC and participants in the mentoring program. This open programmatic review was meant to be consistent with the program’s PAR principles and to support participatory design efforts, which are the hallmark of all CPH-NEW programs.

## 4. Discussion

Programs supporting mentor–mentee relationships, based on consensual health-related goals, are relatively novel in the occupational health literature [32], and rarer still in corrections. In this paper, the process used to design and implement an HMP for newly hired COs is described. The HMP is distinguished by its year-long duration and its Total Worker Health emphasis on work life and well-being, going beyond job task familiarization.

Key to the development of this HMP were the two concepts that define CPH-NEW. These two concepts are PAR methods, based around active participation at the line level in program design and implementation efforts, and adherence to Total Worker Health^®^ principles, which requires an integration of individual health with working conditions and work organization. PAR methods were used to actively engage union leadership, COs, and managers in the design and implementation of Total Worker Health interventions. The presumptive value of incorporating bottom-up workforce knowledge of the stages of adaptation and of pitfalls and barriers was implicit. Another presumption, also consistent with PAR principles, was that correctional employee-driven design and involvement offered a heightened potential for participation, effectiveness, and sustainability [13]. Given its basis in PAR methods and Total Worker Health principles, the HMP is innovative and demonstrates feasibility in future applications in corrections. The future of work should involve careful intervention designs that are specific to unique job settings. Extensive application of both PAR methods and Total Worker Health principles in the design of the HMP enabled the effective development of an intervention that is likely to positively affect COs, whose jobs pose multiple obstacles to health and are resistant to change.

Support for organizational change was maintained through the SWSC. The SWSC provided ongoing program oversight pertaining to allocation of compensated time for training, mentoring, and evaluation, and instructed supervisory staff in program accommodation. As noted, the SWSC had a normalizing function. It assumed the ad hoc introductory work performed by the bargaining unit presidents, the study team, and DOC administration, when the start-up phase was complete. This two-part maturation process grew naturally as the bargaining units pursued initial dominance of design and implementation. It was found that many of the predictions of conflict did not arise, even though the developmental process of the program was largely exclusive of supervisory representation. Whether or not a more direct preliminary action phase is a necessity will likely depend on local, rather than elemental, conditions. However, it is clear that the autonomous contributions of the line workforce, even if temporarily in isolation, are elemental.

More than a decade before the HMP, the DOC had introduced a more traditional but concerted mentoring program which was principally top-down in its implementation. It proved to be unsustainable. The HMP offered a different level of engagement in which line-level staff, union leaders, and DOC management worked together in program design and took responsibility for its continuity. The HMP within HITEC II could not have been, however, a purely bottom-up creation. Bargaining unit leadership is largely consumed in contractual affairs, and program management is a learned and selective skill. Moreover, the constraints over aligned scheduling, coverage, and compensation fall outside of bargaining unit jurisdiction. A high level of programmatic autonomy had been negotiated and maintained through the SWSC. Thus, there was always an institutional readiness to address critical barriers and promote engaged activity. Conversely, a purely line officer program is likely to fail because officers change facilities and have insufficient administrative experience and authority to manage a complex program such as the HMP. The ability of administrators and supervisors to flexibly facilitate, rather than direct, within defined parameters, is essential to program success and has required a sophisticated SWSC.

Additionally, the HMP was part of a research study requiring the support of HITEC II research staff. HITEC II research staff presented a research design and created a battery of process assessment tools that were vetted through the participatory design focus groups and the SWSC, but which would not have evolved without academic engagement. The goal of the HMP program was to help COs develop and maintain positive health behaviors; to provide COs with peer coaching around physical fitness; to prevent early decline in fitness; and to improve stress management. HITEC II research staff also provided support with training mentors, and baseline and follow-up assessments. Further, research staff created a website for COs with customized health information, in addition to a newsletter that was circulated to various facilities on a quarterly basis. The HITEC II research team has placed its methods in the public domain with the development of a toolkit to guide peer mentoring in correctional workforce [33], but it must be conceded that the particular combination of academic, labor union, and administrative interplay is unusual [34]. The HMP with a Total Worker Health approach addresses many components of organizational design at work and beyond for a healthy future of work in corrections by training mentees on outside work factors such as work-family balance.

Certain adaptations also occurred during program implementation. These included changes to mentor eligibility criteria and supervisor involvement. Initially, eligible mentors were required to have had five years of experience or more on the job. However, it become evident early in the program initiation that interested tenured staff at various facilities were in the process of retirement, which resulted in scheduling problems. To increase the pool of mentors interested in the HMP, the eligibility criteria changed from five to three years’ experience, and even less in some cases. It became clear that highly motivated younger officers were capable of superior performance. As the selection process matured, a more personalized approach to admission replaced rigid guidelines, in some cases. Additionally, younger, less tenured COs were considered to suffer from less burnout and could be expected to provide a more positive and healthy perspective on the job. In later program review, there was a realization that the process of development from engaged mentor to health and well-being advocate and champion was continuous, and in the long run may be constructed along continuous improvement lines.

During program design, the supervisors’ role in program implementation was secondary to that of CO leadership. However, during implementation, supervisors played a key role in relieving mentees and mentors from their shift assignments in order for them to meet during work hours. Supervisors also helped recruit mentors, and their possible role in training mentors was also discussed by the SWSC. Ultimately, the supervisors developed their own health promotion programs for supervisors. The traditional division between supervisory and CO staff softened on issues involving health and well-being. The need for changes in program design for continuous improvement played a key role in determining the important function that the SWSC played, especially during implementation of the HMP.

It is also important to highlight some of the barriers that were overcome in the process of developing the mentoring program. First, there was a lack of precedent materials for a CO mentoring program. As such, it was necessary to bring all levels of correctional staff to work together in creating and formatting the training materials. Second, COs have limited formal administrative and/or organizational experience in conducting and evaluating programs. This necessitated union leadership and SWSC engagement, in addition to engagement of study staff to design and conduct this participatory program. Third, problems of vetting and confidentiality during the recruitment of mentors were handled with an elaborate mitigation process that was acceptable to the administration and the union, which initially followed the formal administration-union grievance practices. Over time, actual working relations softened these restrictions. Finally, barriers around varying shift and work slots between mentors and mentees were identified and addressed early during the design and implementation of the mentoring program. Considerations over scheduling meeting time and shift alignments of mentors and mentees were discussed at labor management to maximize the amount of time available for mentors and mentees to meet.

The HMP benefited from being introduced in the context of the ongoing HITEC II study that included efforts to compare two different means of engaging employees in the participatory design of Total Worker Health interventions [1]. Separate employee “design teams” were tested, one consisting of front-line corrections staff only, whereas the other was multi-level and included supervisors and managers. Both design teams engaged in the design and implementation of interventions to address a set of preselected health and wellness issues [1]. The SWSC was formed during the initial phase of the HITEC study to oversee the design teams. Thus, the HMP from its inception could rely on a functioning oversight committee that was well versed and skilled in PAR. Based on these study experiences, CPH-NEW has developed a new toolkit to guide organizations interested in establishing an HMP [33]. Additional guidance on forming and maintaining a dedicated steering committee is available in the CPH-NEW Healthy Workplace Participatory Program Toolkit [35]. Organizations seeking to expand employee involvement in designing Total Worker Health interventions, including implementation of a peer health mentoring program or a Healthy Workplace Participatory Program, are now able to assess key program facilitators and obstacles prior to implementation with an Organizational Readiness Tool survey [36]. The Organizational Readiness Tool assesses organizational readiness for participatory Total Worker Health programs in the following eight domains: (1) Current safety/health/well-being programs; (2) Current organizational approaches to safety/health/well-being; (3) Resources available for safety/health/well-being; (4) Resources and readiness for change initiatives to improve safety/health/well-being; (5) Resources and readiness for use of teams in programmatic initiatives; (6) Teamwork; (7) Resources and readiness for employee participation; and (8) Management communication about safety/health/well-being. In addition to being used for pre-implementation planning, the ORT can be administered multiple times during program implementation to track the extent to which barriers and obstacles to implementation are being fully addressed [37].

Promoting employee efficacy in the design of Total Worker Health interventions and programs such as peer health mentoring has important implications for the future of work. With so many internal and external factors impacting present-day workplaces in ways that critically affect health, including COVID-19 and the accelerating pace of technological change, work organizations must not only respond quickly but also in a manner that draws upon expertise from all levels of their organization. Peer-to-peer programs have gained recognition in addressing job satisfaction and worker health [23,24,38]. However, to our knowledge, this is the first mentoring program focused on health that has been designed for newly hired COs using participatory methods. The empowerment of COs offered through this participatory program to improve the health and safety of their peers is an important progression in creating a healthy correctional workforce, beginning a process of changing correctional culture. Using an approach based on continuous and documented improvement opened the HMP to a long-term view of interventions in which successes, failures, and ongoing operations can be accommodated.

## 5. Conclusions

In the first wave of practice, the preparation proved to be effective because of its reach; nearly 100 mentors were trained, and 269 participants (mentees and controls) signed up and completed baseline testing. Evaluation findings of the HMP are discussed in a separate article within this Special Issue [30].

CO involvement in participatory design efforts can serve to overcome widespread resistance and distrust by COs towards preventive health and work programs. CO involvement could also be effective in alleviating the culture of reluctance by COs to share health and well-being issues of a confidential and personal nature with their peers/DOC staff. In addition to initial planning efforts with union leaders and corrections management, support for the HMP remained high as program evaluation efforts continued, suggesting the likelihood of program sustainability. HMP has shown promise as an intervention within the correctional sector; further evaluation is needed to demonstrate generalizability and adaptability of such programs across state lines and specific correctional settings (such as jails).

## Figures and Tables

**Table 1:** Number and percent of mentees by location, and mentors who volunteered.

Location	Mentees		Mentors	
	Number	Percent	Number	Percent
A	30	16.4%	19	18.1%
B	17	9.3	10	9.5
C	14	7.6	7	6.7
D	23	12.5	14	13.3
E	19	10.4	12	11.4
F	18	9.8	12	11.4
G	7	3.8	2	1.9
H	24	13.1	10	9.5
I	20	10.9	8	7.6
All others (*n* = 4)	11	6.0	11	10.5
Total	183		105	

## Data Availability

Data presented in this study is available on request from the corresponding author. The focus group data are not publicly available due to confidentiality concerns with consent agreement and organizational rules/policies with IRB and DOC.

## Abbreviations

CO	Correctional Officer
HMP	Health Mentoring Program
SFP	Standard Follow-up Program
PFP	Personalized Follow-up Program
NIOSH	National Institute for Occupational Safety and Health
HITEC	Health Improvement Through Employee Control
CPH-NEW	Center for the Promotion of Health in the New England Workplace
PAR	participatory action research
DOC	Department of Correction
SWSC	Study-wide Steering Committee
OJT	On the Job Training
